# The Heme Oxygenase/Biliverdin Reductase System as a Therapeutic Target to Counteract Cellular Senescence in Alzheimer’s Disease

**DOI:** 10.3390/antiox14101237

**Published:** 2025-10-15

**Authors:** Cesare Mancuso

**Affiliations:** 1 Fondazione Policlinico Universitario Agostino Gemelli IRCCS, 00168 Rome, Italy; cesare.mancuso@unicatt.it; 2 Department of Healthcare Surveillance and Bioethics, Section of Pharmacology, Università Cattolica del Sacro Cuore, Largo Francesco Vito, 1, 00168 Rome, Italy

**Keywords:** β-amyloid, bilirubin, biliverdin, carbon monoxide, non-steroidal anti-inflammatory drugs, proliferation signal inhibitors, senolytics, senomorphics, statins

## Abstract

Alzheimer’s disease (AD) is a neurodegenerative disorder involving free radical overload, neuroinflammation, and a deranged cell stress response. In particular, the modulation of the heme oxygenase/biliverdin reductase (HO/BVR) system, a key component of the brain stress response, is currently regarded as a promising therapeutic approach for AD. Cellular senescence, defined as a process of cell cycle arrest due to oxidative stress, DNA damage, mitochondrial dysfunction, and oncogene activation, has been identified as a pivotal factor in the development of AD. A mounting body of research has demonstrated that the accumulation of senescent cells in the brain can lead to a variety of neurotoxic effects, including synaptic dysfunction, the destruction of the blood–brain barrier, and impaired remyelination. Finally, the release of proinflammatory molecules by senescent cells further exacerbates neurodegeneration. A considerable number of xenobiotics, with well-documented neuroprotective effects through the activation of the HO/BVR system, have been shown to modulate pathways involved in cellular senescence outside the brain. Unfortunately, a direct link between HO/BVR and cellular senescence in AD is yet to be established. This compelling evidence should motivate basic and clinical researchers to address such a significant gap in knowledge and conduct novel studies in this field.

## 1. Introduction

In the first century B.C., the renowned Roman rhetorician and philosopher Marcus Tullius Cicero praised old age and its virtues, considering it as a period of wisdom and self-control. However, he specified that this peaceful transition required adopting virtuous lines of behavior and lifestyles during one’s youth [1]. In contrast, approximately a century earlier, the Roman playwright Publius Terentius Aphro had cautioned against the pitfalls of senility, associating the afflictions and deprivations that ensued from a wasteful existence to a frank disease [2]. It is possible to interpret these two philosophical aspects of aging as a metaphor for the physiological processes that take place within cells. Under normal conditions, the cell transits through the proliferation, differentiation or quiescence stages depending on its final commitment. Nevertheless, under stressful conditions, including either redox imbalance or strong inflammation or oncogene activation, the cells may elude their physiological cycle, undergoing stable growth arrest and becoming senescent cells [3]. Among the main features of a senescent cell, it is worth mentioning the remodeling of nuclear chromatin, increased mitochondria activation, the enhancement of proteostasis, and the activation of antiapototic pathways [3,4,5]. In addition, senescent cells can acquire the so-called senescence-associated secretory phenotype (SASP), characterized by an increased release of proinflammatory cytokines, chemokines, proteases and growth factors (Table 1) [3,6,7,8]. While senescence per se is not always a negative status, considering that senescent cells can trigger tissue repair and act as a barrier against tumorigenesis, their accumulation triggers the onset of age-related diseases, such as Alzheimer’s disease (AD) [3,4,5]. In order to counteract cell senescence, several agents have been proposed, and classified into two main categories, “senolytics” and “senomorphics”. Senolytics, which are intended to be used as single-shot agents because of their long-term effects, clear senescent cells by restoring apoptosis or enhancing immune response, whereas senomorphics prevent SASP-induced cytotoxicity and require chronic treatment, which can also give rise to off-target effects [3,4].

**Table 1:** Main components of SASP in neurodegenerative disorders.

SASP Factor(s)
IL-1α, IL-1β
IL-6
IL-8
TNF-α
TGF-β
MMP-3

IL, interleukin; MMP, matrix metallopropteinase; TNF, tumor necrosis factor; TGF, transforming growth factor. For further information, see [5,9,10].

**Figure 1:**
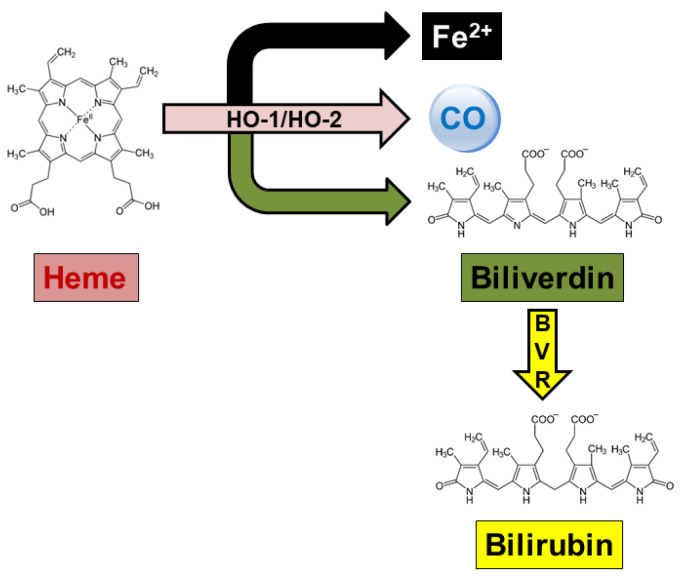
The heme degradation pathway. Heme oxygenase (HO) exists in two isoforms, HO-1 and HO-2. Heme oxygenase-1, inducible under prooxidant and proinflammatory conditions, is almost ubiquitous, but abundant in the liver and spleen where is involved in the degradation of aged red blood cells. Conversely, HO-2, mainly expressed in neurons and testes, is implicated in the physiological turnover of heme and intracellular oxygen sensing. Despite the different distribution and regulation, HO isoforms catalyze the same reaction, namely the oxidation of the α-meso-carbon bridge of heme moieties into ferrous iron (Fe^2+^), carbon monoxide (CO), and biliverdin-IXα . This reaction occurs in the presence of oxygen and reducing equivalents provided by NADPH-cytochrome P-450 reductase, and has NADPH as a cofactor. Once formed through the HO activity, biliverdin-IXα is reduced to bilirubin-IXα by the enzyme biliverdin reductase (BVR). This reaction requires as cofactors either NADH or NADPH at acidic pH or alkaline pH, respectively, and free thiols. Reproduced, with permission, from [12].

**Figure 2:**
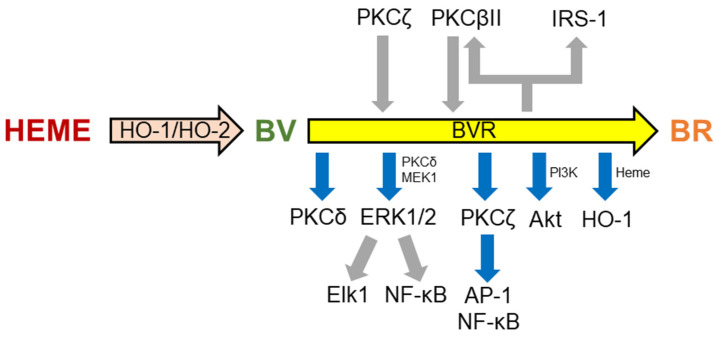
Summary of the main targets of biliverdin reductase (BVR). Upon phosphorylation by IRK-1, BVR phosphorylates IRS-1, thus playing a pivotal role in insulin signaling. In addition, BVR phosphorylates PKCβII, the latter being able to activate BVR under severe conditions for autophosphorylation. As far as the atypical PKCζ and PKCδ is concerned, these isozymes associate with BVR under inflammatory and carcinogenetic stimuli, such as TNF-α and PMA. Through the association with PKCζ, BVR activates proinflammatory transcription factors, such as AP-1 and NF-κB. By forming ternary complexes with either MEK1 and ERK or PKCδ and ERK2, BVR enables the nuclear translocation of ERK1/2 and the activation of Elk1 and NF-κB. Ultimately, BVR activates the PI3K/Akt system and the pro-survival pathways downstream. Gray arrows, phosphorylation; blue arrows, activation or protein/protein interaction. AP-1, activator protein-1; BR, bilirubin-IXα; BV, biliverdin-IXα; ELK, ETS-like-1 protein; ERK, extracellular signal-regulated kinase; IRK-1, insulin receptor kinase-1; IRS-1, insulin receptor substrate-1; MEK, mitogen-activated protein kinase kinase; NF-κB, nuclear factor-κB; PI3K, phosphatidylinositol-3 kinase; PKC, protein kinase C; PMA, phorbol myristate acetate; TNF-α, tumor necrosis factor-α. Reproduced with permission from [15].

Heme oxygenase (HO) metabolizes the hemoprotein heme moieties into ferrous iron, carbon monoxide (CO) and biliverdin-IXα (BV), the latter being reduced into bilirubin-IXα (BR) by biliverdin reductase-A (BVR) [11,12,13,14] (Figure 1). The inducible isoform, HO-1, is almost ubiquitous and undergoes upregulation under prooxidant and/or proinflammatory conditions [14,15,16,17]. As a result, both the HO-1-related decrease in redox-active heme and increase in cytoprotective CO and BV, contribute to the restoration of homeostasis [18,19]. On the contrary, HO-2, the constitutive isozyme, is involved in the physiologic turnover of heme and intracellular oxygen sensing [12,14,20]. Concerning BVR, this pleiotropic enzyme not only converts BV into BR, a strong scavenger of both ROS and RNS, but also, by serving as a serine/threonine/tyrosine kinase, is involved in the regulation of several intracellular pathways [13,15,21,22] (Figure 2). Several studies have described genetic variants of the HO/BVR system. Gain-of-function mutations in the HO-1 and HO-2 genes reduce the risk of coronary heart disease and age-related macular degeneration, but increase the risk of neonatal jaundice, sickle cell anemia, and Parkinson’s disease [15]. Conversely, loss-of-function mutations in the HO-1 gene increase the risk of type 2 diabetes mellitus, chronic obstructive pulmonary disease, and certain types of cancers. Regarding BVR, reported loss-of-function mutations increase the risk of green jaundice [15].

However, in the event of a protracted redox imbalance, such as what occurs in cases of reactive oxygen and nitrogen species (ROS and RNS, respectively) overproduction, both HO-1 and BVR undergo post-translational modifications that result in alterations of their cytoprotective effects. The latter evidence has been supported by studies on brain areas of patients with AD or mild cognitive impairment (MCI), the latter being its prodromal phase [23,24,25].

Over the last decades, several papers have reported the ability of xenobiotics, including drugs [e.g., acetylcholinesterase inhibitors (AChEIs), statins, proliferation signal inhibitors, non-steroidal anti-inflammatory drugs] and natural products (e.g., ferulic acid), to improve neuroprotection and promote healthy aging through the regulation of the HO-1/BVR system [18,26,27,28,29,30]. The ability of CO and the BV/BR pair to activate neuroprotective pathways and down-regulate neurotoxic signals, has made the HO-1/BVR system a promising drug target for AD [18,31]. Furthermore, evidence that most CO/BV/BR targets are involved in the regulation of cellular senescence further increases interest in the HO-1/BVR system at the aging/senescence boundary.

The purpose of this review is not to provide an accurate description of the mechanisms underlying the regulation of either the HO-1/BVR system or cellular senescence. Readers interested in these topics can access many exhaustive and updated articles in the literature. On the other hand, this study aims to discuss the role of the HO-1/BVR system in the regulation of cell senescence and the possible implications for AD.

## 2. Cellular Senescence and Alzheimer’s Disease

The “amyloid cascade hypothesis” theorizes that amyloid-β-peptide (Aβ) plays a pivotal role in the onset and progression of AD. β-amyloid is composed of 36 to 43 amino acids and is produced by the sequential cleavage of the amyloid precursor protein (APP) by β- and γ-secretases [32]. Upon formation, Aβ tends to self-assemble into spontaneous aggregates, such as oligomers or fibrils [33,34,35]. The latter form insoluble secondary structures, which comprise the core of senile (or amyloid) plaques. However, more recently, new scientific evidence has revealed that an imbalance in the production and clearance of soluble Aβ may be an early pathological feature that triggers synaptic dysfunction and cognitive decline in AD [35]. A process known as the unfolded protein response (UPR) or proteostasis, which is ubiquitin-dependent, enables the degradation of both Aβ oligomers and fibrils by neurons, but when this process is not efficient enough, misfolded Aβ accumulates leading to cell senescence [36,37,38]. Another main player in AD pathophysiology is the tau protein that undergoes hyperphosphorylation by specific Aβ-activated kinases, including glycogen synthase kinase-3β (GSK-3β), cyclin-dependent kinase 5 (cdk5), and dual specificity tyrosine phosphorylation regulated kinase 1A (DYRK1A) [39,40,41,42]. Consequently, the hyperphosphorylated tau becomes insoluble, its affinity for microtubules declines, and it forms aggregates with a double-helix secondary structure. As for Aβ, misfolded tau that is not efficiently degraded by the proteasome accumulates and contributes to cell senescence [36,42,43]. The mechanisms underlying the induction of cellular senescence in the AD brain are multifaceted and include free radical production, mitochondrial dysfunction, DNA damage and growth arrest, impaired cell stress response, neuroinflammation, and apoptosis [44,45,46,47,48,49].

A significant increase in the production of ROS, particularly superoxide anion, is undoubtedly related to mitochondrial damage caused by Aβ production and tau hyperphosphorylation [50,51]. This damage is demonstrated by alterations in important enzymes involved in the mitochondrial respiratory chain (e.g., complexes I, II, and IV) and Krebs cycle (α-ketoglutarate and pyruvate dehydrogenases) [42,52,53]. Additionally, the opening of mitochondrial membrane transition pore, and the following cytochrome c release and caspase-3 activation, induce apoptosis [54,55]. Finally, superoxide anion reacts with nitric oxide (NO), overproduced because of nitric oxide synthase (NOS)-2 induction, leading to peroxynitrite formation [56]. Superoxide anion and peroxynitrite are both responsible for the oxidation and nitration of proteins and lipids, which generate protein carbonyls (PC), 4-hydroxy-2-nonenal (4-HNE) and 3-nitrotyrosine (3-NT) protein adducts [57,58]. Oxidative damage is also enhanced due to an insufficient cellular stress response, as demonstrated by the reduced expression of catalase, glutathione peroxidase and peroxiredoxin in the parieto-temporal cortex, basal ganglia, and amygdala of subjects with AD [59,60]. Section 3 will address the role of the HO/BVR system in AD. It is important to acknowledge that ROS can induce cellular senescence through another primary mechanism, namely the induction of DNA damage [61,62,63]. In the event that DNA lesions exceed the DNA damage response pathway, a signaling cascade that triggers repair and permanent cell cycle arrest is activated, leading to senescence [61,64]. The phenomenon of cell cycle arrest is predominantly attributable to the activation of tumor suppressor pathways, including p21/p53 and p16^INK4a^ [61,65]. The upregulation of p53 and its downstream effector p21CIP1, together with an overexpression of p16^INK4a^, a critical component of the G1 checkpoint, inhibit the progression of cell cycle from the G1 to S phase and cause cell senescence [5,66,67,68]. As far as AD is concerned, research conducted on human brain samples and animal models has demonstrated that Aβ and tau protein induce a senescence-like phenotype in both astrocytes and oligodendrocyte cells [5,10,69,70]. This phenotype is characterized by the upregulation of p21/CDKN1A, p16^INK4a^/CDKN2A proteins, and senescence-associated β-galactosidase (β-gal) activity [10,69]. In this situation, the clearance of p16^INK4a^-positive cells has been shown to offer protection from both Aβ and tau pathology, while concomitantly leading to improved cognitive functions in mouse AD models [5,9]. That said, the contribution of p21- and p16-driven pathways to cellular senescence in post-mitotic cells, such as neurons, remains a widely debated and studied issue [4,5,9].

While the inflammatory stimulus triggered by Aβ and tau protein is considered an adaptive response in the early stages of AD, as it can activate glial cells and promote phagocytosis of misfolded proteins, the chronic inflammation, which occurs in the later stages of neurodegeneration, reduces the phagocytic activity of astrocytes and microglia [5]. Under this condition, astrocytes and microglia develop a proinflammatory behavior that exacerbates oxidative damage [5]. The uncontrolled production of proinflammatory cytokines from glial cells has been demonstrated to promote cellular senescence and the induction of a secretory phenotype [5]. In this scenario, oxidative/inflammatory damage potentiates the additional release of proinflammatory cytokines by SASP, thus triggering a vicious cycle that can self-fuel cellular senescence [5]. As previously indicated, the degradation of misfolded Aβ and tau protein is not only at the expense of activated glia, but requires the neuronal ubiquitin-proteasome system. Indeed, both proteostasis and senescence have been shown to share several pathogenic mechanisms, including the activation of the mammalian target of rapamycin (mTOR) [4,5,71]. In line with this observation, it has been demonstrated that the attenuation of altered proteostasis, through the administration of an mTOR inhibitor, alleviates proteotoxic stress and reduce cellular senescence [4,5,71].

Di Domenico et al. [72] demonstrated a remarkable correlation between oxidative damage and proteostasis in brain samples taken from subjects diagnosed with Down syndrome (DS). This condition is associated with an elevated risk of developing AD, because of elevated levels of APP in the brain, resulting from the triplication of chromosome 21. In the frontal cortex of DS patients, protein carbonyl adducts were detected in many components of the intracellular quality control system, such as glucose-regulated protein-78, ubiquitin carboxy-terminal hydrolase L1, vacuolar-type ATPase, cathepsin D and glial fibrillary acidic protein. This oxidative post-translational modification is associated with a decrease in the activity of the proteasome and an increase in the formation of autophagosomes [72].

## 3. Alzheimer’s Disease and the Heme Oxygenase/Biliverdin Reductase System

The early hypothesis concerning the role of the HO-1/BVR system in the pathogenesis of AD was proposed in the mid-1990s, when some Authors described a significant HO-1 overexpression in cortical and hippocampal astrocytes and neurons of subjects with AD [73,74,75]. Subsequent studies supported the pivotal role of HO-1 in AD. Inhibition of HO-1 activity by APP has been demonstrated to promote neurodegeneration in mouse cortical neurons, likely by reducing BR synthesis [76]. Conversely, HO-1 upregulation has been shown to reduce tau protein hyperphosphorylation in M17 neuroblastoma cells by inhibiting the phosphorylation of extracellular signal-regulated kinase (ERK)1/2 [77]. Regarding BVR, its dysfunction, occurring at early age, has been demonstrated to promote insulin resistance, foster BACE1 phosphorylation and increase Aβ production in beagle dogs, a well-known preclinical model of AD [78]. The neuroprotective activity of the HO/BVR system was further supported by growing evidence suggesting that CO and BR play a pivotal role in regulating synaptic plasticity and counteracting free radical damage. In this regard, CO was considered, as NO, a neuromodulator capable of enhancing LTP in the rat hippocampus and was believed to be involved in the acquisition and consolidation phases of memory [27,79,80,81]. As for BR, its role as an antioxidant and free radical scavenger has been proposed as early as the initial studies by Roland Stocker and his group [82]. This hypothesis was subsequently confirmed by Sylvain Dorè and coworkers [83], and more recently, by our own research group [84,85]. Alongside the studies that promoted the neuroprotective effect of HO-1, additional developing research suggested its neurotoxic action. As reported by Hyman Schipper’s group, the upregulation of HO-1 resulted in an increased iron sequestration within the mitochondria of rat astrocytes treated with Aβ [86,87]. This increase in iron sequestration subsequently led to oxidative stress-induced neurotoxicity. As a result of these findings, Gupta and Colleagues demonstrated that pharmacological inhibition of HO-1 activity improved behavioral deficits in the APPswe/PS1E9 mouse model of AD [88].

A significant contribution to the resolution of the dilemma on the neuroprotective or neurotoxic effects of the HO/BVR system in AD was made by a productive collaboration between D. Allan Butterfield’s group and our laboratory. This collaboration was made possible thanks to the research conducted by Eugenio Barone, who was at the time a highly skillful and promising Ph.D. student. A substantial upregulation of HO-1 and BVR was detected in post-mortem hippocampal samples from subjects with AD and MCI [23,24,25]. However, a more detailed examination revealed that both HO-1 and BVR were undergoing phosphorylative, oxidative, and nitrosative post-translational modifications [23,24,25]. These modifications altered their enzymatic activity and interaction with other signaling systems located downstream. Notably, a significant decrease was documented in BR and ERK2 levels within the hippocampus of subjects with AD.

On the whole, the findings summarized in this section support the hypothesis that the neuroprotective versus neurotoxic effects of both HO-1 and BVR are dependent upon the duration and intensity of their expression, as well as the nature of the redox microenvironment. Indeed, among the most common causes of perturbation of the cellular milieu is the overproduction of free radicals, as in chronic oxidative and inflammatory damage. Therefore, the absolute HO-1 levels, and its post-translational modifications induced by ROS/RNS, should be considered as two sides of the same coin. During the early stages of neurodegeneration, HO-1 tends to overexpress in order to counteract oxidative and inflammatory damage. However, if free radical production becomes chronic, oxidative and nitrosative modifications would overcome the ability of HO-1 to upregulate, resulting in significant altered functions.

## 4. The HO/BVR System and Cellular Senescence

With regard to the contribution of the HO/BVR system in the cellular senescence induction, only few studies in the literature have examined this topic, and an even smaller number has focused on brain diseases. In particular, recent studies have examined the role of HO-1 in intracerebral hemorrhage (ICH) sequelae, as well as the mechanisms by which this isoform is involved with radioresistance in glioblastoma multiforme (GBM). Additional evidence regarding the role of both HO-1 and BVR in cellular senescence has been obtained through experimental models associated with cardiovascular and pulmonary diseases, as well as age-related cataract (ARC).

In an ICH experimental model, transient exposure to 10 μM hemin, which resembles the clinical scenario occurring in small bleedings, induced an early senescence-like phenotype in both SH-SY5Y and human induced pluripotent stem cell (iPSC)-derived neurons as well as endothelial cells [89]. This phenotype was characterized by DNA damage, β-gal expression, and NF-κB-mediated HO-1 induction, thus implying that HO-1 is part of a well-regulated machinery [89]. On these grounds, the early phase of senescence can be regarded as an adaptive cellular response to neutralize excess heme through an increase in HO-1-mediated degradation. This response is maintained on condition that there is sufficient availability of storage proteins, such as ferritin, for the scavenging of HO-1 derived free iron [89]. The inhibition of this early senescence, through the blockade of p21, downregulated HO-1 and increased ferroptosis-related lipid peroxidation, suggesting a neuroprotective role for this transient stage against acute cell death by hemin [89]. The evidence that HO-1 inhibition, by a siRNA approach, enhances apoptosis in SH-SY5Y neurons further corroborates the functional neuroprotective role of HO-1 in early senescence. The observation that HO-1 colocalizes with β-gal in neurons surrounding hematomas in brain tissues from ICH patients, further supports the clinical relevance of the inducible isoform in brain cell senescence [89]. In GBM, a highly aggressive brain tumor with a five-year survival rate of about 7%, the accumulation of senescent cells is considered a primary mechanism underlying chemoresistance, radioresistance, and the tendency to recur [90,91]. Incidentally, in GBM and other neoplasms, HO-1 expression has been shown to reduce sensitivity to chemotherapy and promote tumor growth and malignancy [92]. Quite recently, Zhou et al. [93,94] have established two radioresistant GBM cell lines, U251R and Ln229R, characterized by a senescence-like phenotype, as revealed both by p16 and p21 overexpression, elevated β-gal activity, and increased release of SASP factors. Consistently with this phenotype, U251R and Ln229R cells showed a marked activation of a wide array of genes involved in the immune response. Among these genes IFI16 encodes a protein whose role in inducing cellular senescence is now acknowledged [93]. In both U251R and Ln229R cells, IFI16 expression inhibited ferrous iron- and ROS- induced lipid peroxidation and enhanced antioxidant defenses [93]. These features promoted the evasion of ferroptosis, primarily contributing to the radioresistance of GBM. One mechanism through which IFI16 inhibited ferroptosis involves the upregulation of HO-1 through the proto-oncogene JunD and the transcription factor Sp1 [93]. An interesting finding is that glyburide, a sulfonylurea commonly used to treat type 2 diabetes mellitus, interacted with the pyrin domain of IFI16 and has been shown to restore ferroptosis and radiosensitivity in U251R and Ln229R cells [93]. In mice bearing U251R tumors, the combination of radiotherapy and glyburide increased survival of 60% mice, extending it from 26 days to 60 days. This increase was due to the repression of IFI16, HO-1, and GPX4 genes [93].

In preclinical models outside the brain, HO-1 upregulation has been reported to counteract either natural or hydrogen peroxide (50 μM for 3 h)-induced cell senescence in primary cultures of neonatal mouse cardiomyocytes (NCCM) by reducing p16 and p53 levels [95,96]. Heme oxygenase-1 overexpression has been demonstrated to enhance ejection fraction and reduce IL-1, IL-6, and tumor necrosis factor (TNF)-α release, via the senescence-associated secretory phenotype, in a transgenic HO-1^+/+^ aged mouse model [96]. Among the products of the HO/BVR system, CO and BR have been hypothesized as potential explanations for the cardioprotective effects observed. As a matter of fact, pretreatment with the CO-releasing molecule (CORM)-2 (100 μM for 1 h) or BR (20 μM for 12 h) counteracted the increase in p53 and reduced β-gal levels in NCCM treated with hydrogen peroxide [96]. As far as the vascular system is concerned, the HO-1 knock-down promoted cell senescence in HUVEC cells, with a mechanism involving both reduced vascular endothelial growth factor expression and p16^INK4a^ upregulation [97]. This finding supports prior evidence by Luo et al., who demonstrated, in HUVEC cells, that HO-1 overexpression ameliorates endothelial senescence by promoting NO release through the Akt/eNOS pathway [98]. Carbon monoxide (CORM-2, 5–10 mg/kg, intraperitoneal) inhibited p53 and p21 and suppressed cellular senescence in mouse type II alveolar epithelial cells (AECII) exposed to bleomycin, a drug that induces pulmonary fibrosis [99]. Furthermore, CORM-2 treatment resulted in a significant reduction in SASP-related IL-1β, IL-6, and transforming growth factor (TGF)-β in both AECII and bronchoalveolar lavage fluid of bleomycin-treated (5 mg/kg, single oropharyngeal instillation) mice [99].

In human diploid fibroblast cells (HDF), BVR silencing, through the short hairpin RNA (shRNA) technique, increased both β-gal and p53 and p16^INK4a^ and p21 expression, thus suggesting cell cycle arrest [100]. Furthermore, the downregulation of cyclin D1 and CDK4 in shRNA-BVR-treated HDF further supported the important role of the reductase in the cellular senescence regulation [100]. Comparable results have been obtained in lens epithelial cells (LEC) from subjects with ARC. The application of a short interfering RNA (siRNA) molecule directed against BVR resulted in a substantial increase in β-gal staining in LEC, accompanied by a concomitant upregulation of both p21 and p16^INK4a^ [101]. The induction of cellular senescence by siRNAs has been shown to increase the susceptibility of LEC to free radical damage [101]. This finding establishes BVR as a pivotal cytoprotective enzyme against ARC. The mechanisms through which BVR could prevent cellular senescence, and protect LEC against oxidative stress, involve the increased conversion of BV into BR, and the subsequent restoration of mitochondrial function, as well as the upregulation of HO-1, which is necessary for providing BV [101]. In this experimental system, HO-1 induction occurs both directly, through the nuclear translocation of the transcription factor nuclear factor erythroid 2-related factor 2 (Nrf2), and indirectly, through the activation of the pERK1/2 system [101].

## 5. HO/BVR Modulation and Cellular Senescence in AD: A Significant Field for Future Research

When writing this review, the idea was to describe how the modulation of the HO/BVR system could affect cellular senescence in AD brain. However, as specified in previous sections, it is clear that, despite the plethora of evidence implicating cellular senescence in AD, and the well-established role of the HO/BVR system in the mechanisms underlying this dementia, there was a lack of a direct link between HO/BVR-induced modulation of cellular senescence and AD. That said, it is my opinion that the modulation of cellular senescence by the HO/BVR system has the potential to emerge as a significant research domain, aiming to enhance the accessibility of agents that can counteract AD. Therefore, in this section, I will discuss a series of drugs and dietary supplements for which the modulation of the HO/BVR system has demonstrated an effective neuroprotective effect in AD. These xenobiotics share the ability to regulate signal pathways involved in cellular senescence, suggesting their potential as promising senolytic or senomorphic agents.

### 5.1. Acetylcholinesterase Inhibitors

Acetylcholine (ACh) receptor agonists and cholinesterase inhibitors (AChEIs) are an important group of agents with therapeutic efficacy on the nervous system. The classification of cholinergic stimulants is based on two criteria: their spectrum of action, which is reliant on the type of receptor (muscarinic or nicotinic) activated, and the mechanism of action [18]. In this regard, some cholinomimetics bind and activate cholinoceptors, whereas others inhibit cholinesterase, thus increasing the bioavailability of acetylcholine in the synaptic cleft [18]. Galantamine and donepezil, two AChEIs, are currently used for treating mild AD. In vitro experiments have shown that these drugs, at low micromolar concentrations, decrease cellular senescence in U87 astrocytoma cells that have been exposed to Aβ and in differentiated PC12 cells that have been treated with glutamate [102,103]. The neuroprotective effects of donepezil were enhanced in cases of co-administration with memantine, a non-competitive NMDA receptor inhibitor [103]. In both of these cell lines, the AChEIs reversed cellular senescence, as demonstrated by alterations in cell morphology and β-gal staining, by counteracting free radical production and restoring physiological levels of p53 [102,103]. In an in vivo model of AD, such as the SAMP8 mouse, the combination of donepezil (0.3 mg/kg subcutaneous) and memantine (10 mg/kg subcutaneous) demonstrated a significant reduction in Aβ-induced β-gal staining in the CA1 and CA3 regions of the hippocampus, accompanied by an increase in ACh levels [103]. The neuroprotective effects of AChEIs were not limited to neural cells. As demonstrated by Zhang et al. [104], donepezil (10–50 μM) reversed the senescence of HUVEC cells exposed to elevated glucose levels. The drug reduced ROS generation, down-regulated p21 intracellular levels, and enhanced SIRT1 activity [104]. These effects led to a delay in high glucose-induced cellular senescence. In this situation, galantamine exhibited neuroprotective effects by modulating the HO-1/CO pathway [105]. In primary cultures of cerebral microvascular endothelial cells, 1 μM galantamine was found to upregulate HO-1 and reduce 0.3 mM hydrogen peroxide-induced cell death [105]. These neuroprotective effects were reversed by the inhibition of either NF-κB signaling or HO activity [105]. It is noteworthy that CO (250 parts per million) exerted a permissive effect on galantamine-related neuroprotection in this experimental system [105].

### 5.2. Hydroxy-Methylglutaryl-Coenzyme a Inhibitors (Statins)

Statins are a family of drugs with pleiotropic functions. They block the conversion of HMG-CoA into mevalonate by inhibiting HMG-CoA reductase, which is the first step in cholesterol biosynthesis [106,107]. Consequently, the synthesis of low-density lipoprotein cholesterol (LDL-C) decreases in hepatocytes, which leads to a reduction in blood cholesterol levels. Additionally, statins reduce triglycerides and increase high-density lipoprotein cholesterol (HDL-C) levels in the blood [30]. Along with these metabolic effects, statins also prevent isoprenoid synthesis, such as farnesyl pyrophosphate (FPP) and geranylgeranyl pyrophosphate (GGPP), by reducing mevalonic acid formation. Both FPP and GGPP are required to enable proper subcellular localization and trafficking of intracellular proteins [30,108,109]. In addition, small GTP-binding proteins, including members of the Ras and Rho GTPase family, require prenylation post-translational modifications to function as modulators of the actin cytoskeleton and to participate in intracellular signaling [30,109]. Downstream consequences of reduced isoprenoid synthesis may also include modulation of the insulin/phosphatidylinositol-3-kinase (PI3K)/protein kinase B (Akt) pathway and reduction in free radical production [110,111]. Importantly, the reduction in protein isoprenylation mediated by statins has extensive effects on cellular senescence. As demonstrated by Assmus et al. [112], by preventing isoprenoid synthesis, 0.1 μM atorvastatin inhibited endothelial progenitor cell (EPC) senescence, occurring in case of prolonged cultivation, as demonstrated by a significant reduction in β-gal staining. The mechanism through which atorvastatin inhibits EPC senescence involves the PI3K/Akt-dependent modulation of cell cycle regulatory genes. Indeed, atorvastatin increased the expression of cyclin A and cyclin F, concomitantly reducing p27 expression [112]. The PI3K/Akt axis has been shown to contribute to statin-related modulation of cellular senescence in HUVEC cells [113]. As indicated by Ota et al. [113], atorvastatin (10–100 nM) counteracts hydrogen peroxide-induced HUVEC senescence by increasing the phosphorylation of Akt at Ser473. In this experimental system, Akt phosphorylation activated downstream eNOS, SIRT1, and catalase, three major players involved in cellular senescence [113].

The protective role of statins in preventing or slowing down AD has long been debated. A recent meta-analysis, which included 42 cohort studies with 6,325,740 patients, concluded that the use of statin is associated with an age-dependent reduction in AD incidence [114]. Preclinical and clinical evidence shows that statins have neuroprotective effects in AD by lowering cholesterol, reducing free radical generation, enhancing eNOS, improving cerebral blood flow, and modulating matrix metalloproteinase activity [115,116]. Regarding the role of the cellular stress response in statin neuroprotection, prominent findings have appeared from studies on aged beagle dogs, a prevalent preclinical model of AD due to their deposition of endogenous Aβ with an identical sequence to humans [117]. Long-term atorvastatin treatment (80 mg/day for 14.5 months) increased the expression of the HO-1/BVR system, as well as the phosphorylation of BVR at specific tyrosine residues in the parietal cortex of the treated dogs [29,118]. The latter is a post-translational modification that increases reductase activity. Increased HO-1/BVR expression and activity correlated with a significant reduction in oxidative and nitrosative stress biomarkers in the parietal cortex of aged canines [29,118]. Notably, HO-1 and BVR upregulation improved memory in atorvastatin-treated dogs, as proven by a significant correlation with lower discrimination learning error scores, and reduced BACE1 protein levels [29,118].

### 5.3. Non-Steroidal Anti-Inflammatory Drugs

Nonsteroidal anti-inflammatory drugs (NSAIDs) suppress inflammation through the inhibition of prostaglandin (PG) synthesis via the cyclooxygenase (COX) pathway. This enzyme, also known as prostaglandin PG-endoperoxide synthase, catalyzes two sequential chemical reactions in separate, but functionally coupled, active sites. The COX activity is responsible for the synthesis of PGG2 from two molecules of oxygen and a molecule of arachidonic acid. In contrast, the peroxidase activity catalyzes a net two electron reduction in the 15-hydroperoxyl group of PGG2, yielding PGH2, with the release of an oxidizing radical [119,120,121]. Finally, the transformation of PGH2 into PGE2, PGD2, PGF2, and PGI2 is catalyzed by tissue-specific isomerases [119,121]. Two COX isoforms have been described: the constitutive COX-1, which generates prostanoids for housekeeping functions, such as gastric epithelial protection and platelet aggregation, and the inducible COX-2, an early gene that is upregulated by inflammatory cytokines and growth factors [122]. However, this distinction does not appear as straightforward when considering the central nervous system. Indeed, COX-2 is constitutively expressed in neurons and involved in the regulation of physiological processes, such as neuronal plasticity and anxiety [123,124,125]. In contrast, COX-1 can be induced by neuroinflammatory stimuli, including Aβ and prion protein, which prompts the production of prostaglandins (e.g., PGD2, PGE2) that contribute to the amplification of the inflammatory response [123,124,125].

With regard to the modulation of cellular senescence by COX isozymes and related PGs, the available data vary according to the experimental model. Cyclooxygenase-2 has been demonstrated to enhance the expression of SASP components (e.g., IL-1β, IL-8 and colony stimulating factor) through an autocrine feedback loop involving its downstream product PGE2, which binds to EP4 receptors in both IMR90 lung fibroblasts and mouse hepatocytes [126]. In these experimental systems, COX-2 upregulation occurred through a mechanism involving NF-κB, a key player in the induction of cellular senescence. As demonstrated by Cormenier et al. [127], endoplasmic stress-induced UPR enhanced COX-2 expression through the ATF6α transcription factor in normal human fibroblasts. Consequently, COX-2 upregulation led to an increase in PGE2 synthesis, which triggered cellular senescence by binding to the EP3 receptor [127]. These results support earlier findings that implicated the COX-2/PGE2/EP3 receptor system in the process of cellular senescence in human fibroblasts [128,129]. In a recent study, Wang et al. have reported that midazolam (10–20 μM) alleviated the Aβ-induced β-gal staining as well as p53 and p21 expression in SH-SY5Y neurons by repressing COX-2 expression and the related synthesis of PGE2 [130]. While PGs are considered a primary effector of the COX-2-mediated regulation of cellular senescence, other lines of evidence suggest the involvement of alternate signaling pathways. Acetylsalicylic acid (ASA, 0.02 g/100 mL per os for 7–9 weeks), a prototypical cyclooxygenase-1 (COX-1) inhibitor, has been demonstrated to reverse cellular senescence in the liver, spleen, pancreas, and lung tissues of C57BL/6 mice exposed to doxorubicin (DOX, 1.25 mg/kg intraperitoneal weekly for 4 weeks) during their juvenile stage [131]. Analogous outcomes have been attained in DOX-treated MRC5 human fibroblasts. The underlying mechanism of this protective effect involves a substantial reduction in both p53 and p21 protein levels [131]. The delayed onset of senescence induced by ASA has been confirmed in aged human endothelial cells. However, in these cells 100 μM ASA prevented cellular senescence by activating the NO/cGMP system [132]. In contrast, the acceleration of the senescence of colorectal cancer (CRC) cells by 500 μM ASA was found to be associated with a mechanism involving SIRT1 and phospho-AMPK upregulation [133]. Wang et al. demonstrated that exposure of human chondrocytes to TNF-α resulted in the induction of cell senescence [134]. This was shown by an increase in β-gal staining, cell cycle arrest in the G0/G1 phase, reduced telomerase activity, and increased expression of p21 and p53 proteins [134]. Notably, celecoxib, a selective COX-2 inhibitor, at the concentrations of 10 μM and 20 μM, significantly reversed these biomarkers associated with cellular senescence in human chondrocytes [134]. An analogous phenomenon has been observed in tendon-derived stem cells isolated from patients diagnosed with rotator cuff tendinopathy [135]. In this experimental system, 12.5 μM celecoxib was observed to decrease β-gal staining, alleviate both p16^INK4A^ and p21^CIP1A^ overexpression, and restore physiological p-p65 levels [135].

As far as the involvement in neurodegeneration is concerned, the contribution of COX-1 seems to be insignificant. An absolute increase in COX-1 immunoreactivity has been detected only in cortical microglia (layers II and III) and few neuronal cells [136,137]. Conversely, COX-2 expression has been detected in the perikarions, dendrites and axons of neurons located in the hippocampus (CA1-CA4 subdivision of the pyramid layer), enthorinal cortex, temporal cortex, and frontal cortex of AD patients [136,138,139,140,141,142]. Furthermore, COX-2 upregulation has been detected in both the blood and cerebrospinal fluid (CSF) of subjects with AD and MCI. Higher levels of COX-2 are observed in MCI, and a decline in COX-2 expression is detected as the disease progresses [143]. These last findings, along with the evidence that elevated levels of proinflammatory PGD2 and PGF2α were detected in both blood and CSF samples from MCI patients, supports the hypothesis of an early involvement of COX-2 and its products, making this enzyme a potential biomarker for early diagnosis of AD [144,145]. In particular, PGF2α promoted the transition from MCI to AD by increasing the phosphorylation of tau protein in APOE ε4 carriers [144]. In addition to PG production, COX-2 plays a pivotal role in the progression of AD by modulating the NF-κB and glycogen synthase kinase-3β (GSK3β) pathways. As demonstrated by Cao et al. [146], calcium overload, a prevalent outcome in AD neurons, induced COX-2 and PGE2 synthesis through the activation of NF-κB in tauP301S and COX-2 transgenic mice or neuroblastoma n2a cells. In this experimental system, by binding its own receptor subtypes EP1-3, PGE2 stimulated GSK-3β kinase, which, in turn, enhanced tau protein phosphorylation [146].

Among the alternate pathways through which COX is involved in the pathogenesis of AD, HO is particularly interesting. Heme oxygenase has been demonstrated to modulate COX activity through two principal mechanisms: the reduction in intracellular heme content and the generation of CO [119]. Given that COX is a hemoprotein, heme is an essential cofactor necessary for catalytic activity [147]. Therefore, heme depletion, due to increased HO activity, results in reduced PG release. However, HO-derived CO has been demonstrated to enhance COX activity and PG synthesis in the rat hypothalamus, resulting in an increased release of IL-1β [148,149]. That said, an increase in COX activity, as during inflammation, resulting in elevated levels of PGs and free radicals, may serve as a trigger in the brain for the induction of HO-1 that, in turn, increases cellular antioxidant defenses and counteracts oxidative damage [119]. The functional interplay between these two hemoproteins, prompted our research group to investigate whether NSAIDs, in particular COXIBs, were able to regulate the expression of the HO/BVR system in a preclinical model of AD. As demonstrated by Mhillaj et al. [150], celecoxib (1–10 μM), namely at concentrations which resemble those found in the AD brain after therapeutic doses, increased HO-1 expression in SH-SY5Y neurons exposed to either soluble or fibrillary Aβ [150]. The celecoxib-mediated HO-1 upregulation in Aβ-treated SH-SY5Y was accompanied by a significant reduction in lipid peroxidation damage [150]. Ultimately, among the products of the HO/BVR activities, CO and BR had neuroprotective effects because they prevented ROS production and fostered both the slowdown of the Aβ oligomer growth rate and the decrease in oligomer/fibril final size [150].

Considerably, while the preclinical evidence summarized above has indicated the significance of COX-2 in the development and progression of AD, clinical studies failed to validate this hypothesis. A recent systematic review on this topic, which included four randomized controlled trials (RCTs) with 23,187 participants, has shown that neither ASA nor other NSAIDs, including COXIBs, have been able to prevent AD [151]. Rather, there was some evidence of harm in the groups treated with these drugs [151]. However, the conclusions of this systematic review do not definitively prove the ineffectiveness of COXIBs in AD, because of the limited number of clinical trials and subjects enrolled (1545). Furthermore, the high incidence of adverse effects observed in the groups receiving celecoxib and rofecoxib, the latter no longer available on the market, may be attributable to the prolonged period of treatment with COXIBs.

### 5.4. Proliferation Signal Inhibitors

Proliferation signal inhibitors, such as sirolimus (rapamycin) and everolimus, are a novel class of immunosuppressive agents that are used alone or in combination to prevent the rejection of solid organ allografts [152]. After binding to FKBP12, sirolimus and everolimus block mTOR and the signaling cascade downstream, including the ribosomal S6 kinase (S6K1) and the eukaryotic translation initiation factor 4E-binding protein (4E-BP) [152]. Consequently, both IL-driven T-cell and B-cell proliferation is inhibited along with immunoglobulin production [152]. As previously mentioned, mTOR plays a significant role in cellular senescence by impairing proteostasis, which leads to the accumulation of misfolded proteins, suppressing autophagy, and promoting SASP [4,5]. Remarkably, mTOR upregulation colocalizes with neurofibrillary tangles and modulates tau phosphorylation [153]. Excessive zinc release by presynaptic neurons is a common feature of AD, leading to protein phosphatase 2A inhibition, metabotropic receptor activation, and free radical overload [153,154,155,156]. This exacerbates cognitive dysfunction and memory loss. Zinc treatment has been shown to increase tau protein hyperphosphorylation at Ser356 in the rat hippocampus, as well as downregulate HO-1 expression in this brain area [153]. Interestingly, rapamycin pretreatment (1.5 mg/kg body weight intraperitoneal, three times daily for 1 week) abolished tau protein hyperphosphorylation, HO-1 suppression, and free radical–induced lipid peroxidation and DNA oxidation [153]. In the same experimental system, rapamycin rescued impaired spatial learning and memory functions in zinc-treated rats [153]. Whether HO-1 is an innocent bystander in zinc-induced tauopathy or plays a functional neuroprotective role still remains uncertain. Modulating HO activity using compounds with agonist or antagonist functions (e.g., CoPP-IX or SnPP-IX, respectively) could unravel this mystery.

### 5.5. Ferulic Acid

Ferulic acid, a phenolic acid derivative, is found in high concentrations in various fruits and vegetables, as well as in certain Chinese medicinal herbs (e.g., *Angelica sinensis*, *Cimicifuga racemosa*, and *Ligusticum chuangxiong*) [157,158]. In recent years, a number of studies have demonstrated that FA exhibits a strong antioxidant effect, acting not only through a direct free radical-scavenging mechanism, but also indirectly, by amplifying the cell stress response [157,158]. This antioxidant effect, markedly contributes to the FA’s counteraction of cellular senescence. The exposure to ultraviolet A radiation (UVA) increased intracellular ROS production and decreased both SOD1 and catalase mRNA levels in HDF [159]. This redox imbalance, along with a substantial β-gal staining and an increase in both p16 and p21 mRNA levels, resulting in G1-phase arrest, supported the hypothesis of a noticeable shift to senescence in UVA-exposed HDF [159]. Interestingly, FA (10–20 μM) pretreatment decreased ROS generation and enhanced both SOD1 and catalase levels, thus fostering free radical degradation [159]. Furthermore, FA inhibited the expression of p16 and p21 genes, diminished β-gal staining, and promoted cell cycle progression in UVA-exposed HDF [159]. Moreover, FA has been shown to neutralize cellular senescence through additional mechanisms, such as the upregulation of Sirt1 and AMP-activated kinase (AMPK) [160]. Sirt1, a class III deacetylase, has been demonstrated to reduce inflammation and ferroptosis, by reversing the acetylation of p65 on Lys310, a process required for the optimal transactivation activity of the NF-κB complex, in HDF and rat hippocampal neurons exposed to hydrogen peroxide and oxygen-glucose deprivation, respectively [160,161,162]. In addition, Sirt1 activators have been observed to inhibit TGF-β-activated kinase 1 (TAK1), thereby impeding the activation of both NF-κB and stress-induced p38 and c-Jun N-terminal kinase in murine macrophages infected with *M. tuberculosis* [160,163]. With regard to AMPK, this kinase has been shown to enhance Sirt1 by upregulating nicotinamide phosphoribosyltransferase. The latter, is a rate-limiting enzyme necessary for the regeneration of NAD^+^, a substrate for Sirt1, and for lowering cellular nicotinamide, which has been demonstrated to inhibit Sirt1 activity [160]. Together, these findings provide evidence of an active role of FA in preventing cellular senescence through multiple mechanisms, including free radical degradation, the restoration of cell cycle regulatory proteins to physiological levels, and Sirt1-mediated inhibition of inflammatory response and apoptotic cell death. Finally, and not less important, Sirt1 promotes the nuclear translocation of Nrf2, the transcription factor involved in the expression of HO-1 and other genes associated with the cell stress response [164].

The regulation of the HO/BVR system is an important mechanism involved in FA-related neuroprotection. Ferulic acid and its ethyl ester (FAEE), either at concentrations of 5–50 μM or at the dose of 150 mg/kg body weight intraperitoneal, have been observed to induce the expression of HO-1, and reduce protein oxidation and lipid peroxidation, in rat neurons and gerbil brain synaptosomes exposed to hydroxyl and peroxyl radical damage [165,166,167]. Furthermore, FA (1–10 μM or 150 mg/kg intraperitoneal for 4 days)-induced HO-1 overexpression has been demonstrated to counteract lipid peroxidation-induced damage and reduce apoptosis in both trimethyltin (TMT)-treated SH-SY5Y cells and in hair cells of guinea pigs exposed to acoustic damage [28,168]. The translocation of the transcriptional inducer Nrf2 from the cytosol to the nucleus has been described as the molecular mechanism underlying the FA-induced HO-1 upregulation [28]. Lastly, studies have shown that FA has a neuroprotective effect in psychosocial stress. The administration of FA (150 mg/kg intraperitoneal) to rats exposed to novelty-induced emotional arousal resulted in enhanced long-term memory, which was accompanied by the upregulation of both HO-1 and HO-2 in the hippocampus and frontal cortex [27]. Both the antioxidant and the nootropic effects of FA are attributable to CO and BR, while BV exerts minimal influence on these phenomena [27,28].

As far as the neuroprotective effect of FA in AD is concerned, studies conducted by Allan Butterfield group reported that FAEE (25 μM or 150 mg/kg intraperitoneal) prevented Aβ(1-42)-induced cytotoxicity, ROS generation, protein and lipid oxidation, and induction of inducible nitric oxide synthase in rat cortical neuronal cultures and gerbil brain synaptosomes [169,170]. Cell stress response enhancement appears to cause the neuroprotective effect. This is highlighted by the upregulation of HO-1 and Hsp70 levels in rat cortical neuronals and gerbil brain synaptosomes by FAEE [169,170].

## 6. Conclusions and Future Perspectives

Readers who have persisted to this point in the review may have noticed an unusual paradox: the absence of evidence linking modulation of the HO/BVR system with cellular senescence in AD. This underestimation is challenging for two reasons. Firstly, it hinders further investigation into the signaling pathways that link the HO/BVR system to cellular senescence. Secondly, it reduces the potential for identifying new disease-modifying agents against AD. Recent findings have provided novel insights into the effectiveness of Bcl-2/Bcl-xL/Bcl-W inhibitors, such as navitoclax, in counteracting cellular senescence in AD [4]. In aged wild-type (WT) mice, navitoclax has been shown to eliminate senescent neural precursor cells, enhance the proliferation of hippocampal neural progenitor cells, and improve spatial memory [171,172]. Interestingly, research has shown that venetoclax, which shares the same targets with navitoclax, downregulates antioxidant HO-1, thus increasing ROS levels and apoptosis [173]. Similarly, dasatinib, a Src inhibitor approved for the treatment of leukemia, when administered in combination with quercetin, a natural product that inhibits PI3K, leads to a reduction in senescent oligodendrocyte precursor cells (OPC), a decrease in Aβ, and an improvement in cognition in the APP/PS1 mouse model [69,171]. It is worth noting that dasatinib regulates HO-1 expression in LPS-challenged mouse macrophages [4,174]. Several phase I trials are currently underway to investigate dasatinib safety in patients with AD and MCI. These trials will serve as groundwork for subsequent phase II-III studies, which are necessary to validate this senolytic treatment effectiveness in AD [175,176].

In expressing gratitude to the readers for their interest in this review, the hope is that demonstrating the advantages of further investigation on the association between the HO/BVR system and cellular senescence has been successful. This relationship is particularly interesting due to its various implications from a translational point of view. It is evident that the realization of this goal lies upon active collaboration among specialists in various fields, including biochemists, neurologists, and pharmacologists.

## Data Availability

No new data were created or analyzed in this study. Data sharing is not applicable to this article.
